# Oral amoxicillin treatment disrupts the gut microbiome and metabolome without interfering with luminal redox potential in the intestine of Wistar Han rats

**DOI:** 10.1093/femsec/fiaf003

**Published:** 2025-01-08

**Authors:** Sandra Bermúdez-Sánchez, Martin Iain Bahl, Egon Bech Hansen, Tine Rask Licht, Martin Frederik Laursen

**Affiliations:** National Food Institute, Technical University of Denmark, Kemitorvet, 2800 Kongens Lyngby, Denmark; National Food Institute, Technical University of Denmark, Kemitorvet, 2800 Kongens Lyngby, Denmark; National Food Institute, Technical University of Denmark, Kemitorvet, 2800 Kongens Lyngby, Denmark; National Food Institute, Technical University of Denmark, Kemitorvet, 2800 Kongens Lyngby, Denmark; National Food Institute, Technical University of Denmark, Kemitorvet, 2800 Kongens Lyngby, Denmark

**Keywords:** gut microbiome, antibiotics, redox potential, antioxidant capacity, archaea, SCFAs

## Abstract

Oral antibiotic treatment is well known to be one of the main factors affecting gut microbiota composition by altering bacterial diversity. It decreases the abundance of butyrate-producing bacteria such as *Lachnospiraceae* and *Ruminococcaceae*, while increasing abundance of *Enterobacteriaceae*. The recovery time of commensal bacteria post-antibiotic treatment varies among individuals, and often, complete recovery is not achieved. Recently, gut microbiota disruption has been associated with increased gut oxygen levels and higher redox potential in faecal samples. Given that redox balance is crucial for microbial metabolism and gut health, influencing fermentation processes and maintaining anaerobic conditions, we investigated the impact of oral amoxicillin treatment on the redox potential in the caecum. We used 24 Wistar Han male rats and measured caecal redox potential *in situ* with a probe, before and after 7 days of amoxicillin treatment, as well as after 7 days of recovery. Additionally, we analysed caecal weight, pH, antioxidant capacity, caecal microbiota, metabolome, and colonic tissue expression of relevant genes involved in the redox potential state. Our findings show that oral amoxicillin treatment significantly reduced archaeal load, and decreased the bacterial alpha diversity and affected bacterial composition of the caecal microbiome. The caecal metabolome was also significantly affected, exemplified by reduced amounts of short chain fatty acids during amoxicillin treatment. While the caecal metabolome fully recovered 7 days post amoxicillin treatment, the microbiome did not fully recover within this time frame. However, amoxicillin did not lead to an increase in luminal redox potential in the cecum during or post amoxicillin treatment. Limited differences were observed for colonic expression of genes involved in intestinal barrier function and generation of reactive oxygen species, except for the catalase gene, which was significantly upregulated post-amoxicillin treatment. Our results suggest that while oral amoxicillin disrupts the gut microbiome and metabolome, it does not directly interfere with gut luminal redox state.

## Teaser

Oral amoxicillin disrupts gut microbiome and metabolome without altering luminal redox potential.

## Introduction

Oral antibiotic intake is one of the main disruptors of the gut microbiota, inhibiting the growth of or killing bacteria already from the first day of treatment (Duan et al. 2022). Amoxicillin is a beta-lactam antibiotic with a broad spectrum of activity, effective against many Gram-negative and Gram-positive bacteria. It is one of the most widely prescribed antibiotics and has been strongly associated with gastrointestinal side effects (Pulcini et al. 2017, Huttner et al. 2020). Usually, symptoms such as diarrhoea are transient; however, there is a risk that the gut homeostasis established by the symbiosis between gut bacteria and the host could be irreversibly altered. Consequently, the gut microbiota may not return completely to the initial and highly diverse composition (Ribeiro et al. 2020), often leading to a gut microbiota characterized by an increased abundance of the *Enterobacteriaceae* family (Zimmermann and Curtis 2019) and the depletion of many species within the butyrate-producing bacterial families *Lachnospiraceae* and *Ruminococcaceae*. This has detrimental effects in the gut, due to that butyrate-producing microbial communities are essential for maintaining a healthy environment by preventing the entry and establishment of other microbes, particularly pathogenic ones. Colonocytes utilize butyrate to generate energy, which increases epithelial oxygen consumption (Litvak et al. 2018). This process helps maintain an anaerobic environment in the gut, thereby inhibiting the colonization of opportunistic aerobic pathogens, such as *Salmonella* spp. (Rivera-Chávez et al. 2016) and *Escherichia coli* (Venegas et al. 2019). The resulting compromised commensal microbiota after antibiotic treatment may have consequences for host health, e.g. the expansion of bacteria that belong to the *Enterobacteriaceae* family have been found in inflammatory bowel diseases both in animal models and in humans (Cani 2017, Zuo and Ng 2018).

To improve gut homeostasis during the recovery period after antibiotic treatment and ensure restoration of the healthy microbiome, it is necessary to understand how antibiotics affect the gut microbiota, intestinal abiotic factors, and the host intestinal environment. It has been observed that oral intake of antibiotics affects epithelial O_2_ consumption, and decreases the hypoxia inducible factor 1-alpha (HIF-1α) expression in the colon (Kelly et al. 2015). HIF-1α is expressed under decreased oxygen availability, and its responsible of keeping gut homeostasis by activating transcription of genes involved in barrier integrity protection and regulating bacteria–host interaction (Kumar et al. 2020, Pral et al. 2021).

Recently, a study showed that antibiotics increased *ex vivo* faecal redox potential (Eh) (Rivera-Chávez et al. 2017, Reese et al. 2018). The redox potential represents the overall reducing or oxidizing capacity within a system, which depends on the intestinal oxidative stress, and therefore on reactive oxygen species (ROS), and reactive nitrogen species (RNS) generation. Host immune, epithelial cells, and gut microbiota respond and generate redox signals to keep homeostasis and in response to potential pathogens (Campbell and Colgan 2019). In the host, ROS is generated in the mitochondria of cells via oxidative phosphorylation, mucosal enzymes and through NADPH oxidases such as NOX and DUOX (Sies and Jones 2020). On the other hand, the host and the gut bacteria have mechanisms to cope with the oxidizing molecules, such as the expression of ROS scavenging enzymes: superoxide dismutase, catalase, glutathione peroxidase, etc., thiol groups, and other molecules such as glutathione, uric acid, and vitamins (Nishikawa et al. 2009, Feng and Wang 2020). Previous studies have identified a link between the redox potential and the gut microbiome (Million and Raoult 2018), also describing that changes in redox potential influenced by diet led to compositional shifts in the rumen archaeal community, specifically affecting the abundance of methanogenic archaea (Friedman et al. 2017). However, the redox biology of the human gut microbiota and interaction with the host is not completely understood (Circu and Aw 2012).

Since it may pave the way for new treatment strategies, it is important to know the redox potential and antioxidant capacity of the gut under different conditions, and whether or not it is affected by the gut microbiome composition and the recovery process after its disruption (Albenberg et al. 2014). The role of ROS has been studied in inflammatory gastrointestinal diseases such as inflammatory bowel disease (IBD), ileitis, gastric ulcers, colon cancer, etc. (Aviello and Knaus 2017). In addition, it has been demonstrated that host inflammation responses observed after antibiotic treatment and the subsequent colonization of antibiotic-associated pathogens can generate electron acceptors and other redox-active molecules, leading to oxidative stress (Winter et al. 2010, Spees et al. 2013, Faber et al. 2016). However, there is scarce knowledge about the redox potential in the gut after oral antibiotic treatment. Although an increased redox potential in faecal samples after antibiotic delivery has been reported (Reese et al. 2018), no measurements were made inside the intestinal compartment in this study. To our knowledge the current study is the first to investigate redox potential within the cecum of rats after oral amoxicillin administration. We chose the cecum as the primary site for our analysis because it is a crucial part of the rat's large intestine, playing key roles in microbial fermentation (Xu et al. 2020).

We hypothesize that antibiotic induced microbial disruption in the gut contributes to an increased redox potential within the gut, and in the gut lumen (Spees et al. 2013). In this animal trial, we investigate the impact of oral administration of amoxicillin on redox potential and antioxidant capacity, archaeal load, expression of genes involved directly and indirectly in the production of ROS (NADPH oxidases, ROS scavenging enzymes), and genes associated with hypoxia levels in the gut (HIF-1α), to elucidate the impact of antibiotics on the intestinal health.

## Materials and methods

### Animals

Male Wistar Han rats from Charles River (weight range: 206–253 g) were housed in Makrolon cages and kept at a 12 h light:dark cycle, at a temperature of 22 ± 1°C and relative humidity of 55 ± 5%. Rats were observed twice daily and clinical signs were recorded. The rats were fed a complete breeding-purified diet AIN-93 G (Altromin, Lage, Germany) for the first study and SAFE Scientific Diet A30 (SAFE, Augy, France) for the second animal study. Diet and water were given *ad libitum*. Both animal studies were approved by the Danish Animal Experiments Inspectorate with the authorization number 2020-15-0201-00484-C1. The experiments were overseen by the National Food Institute's in-house Animal Welfare Committee for animal care and use.

### First animal experiment

**Figure 1.**
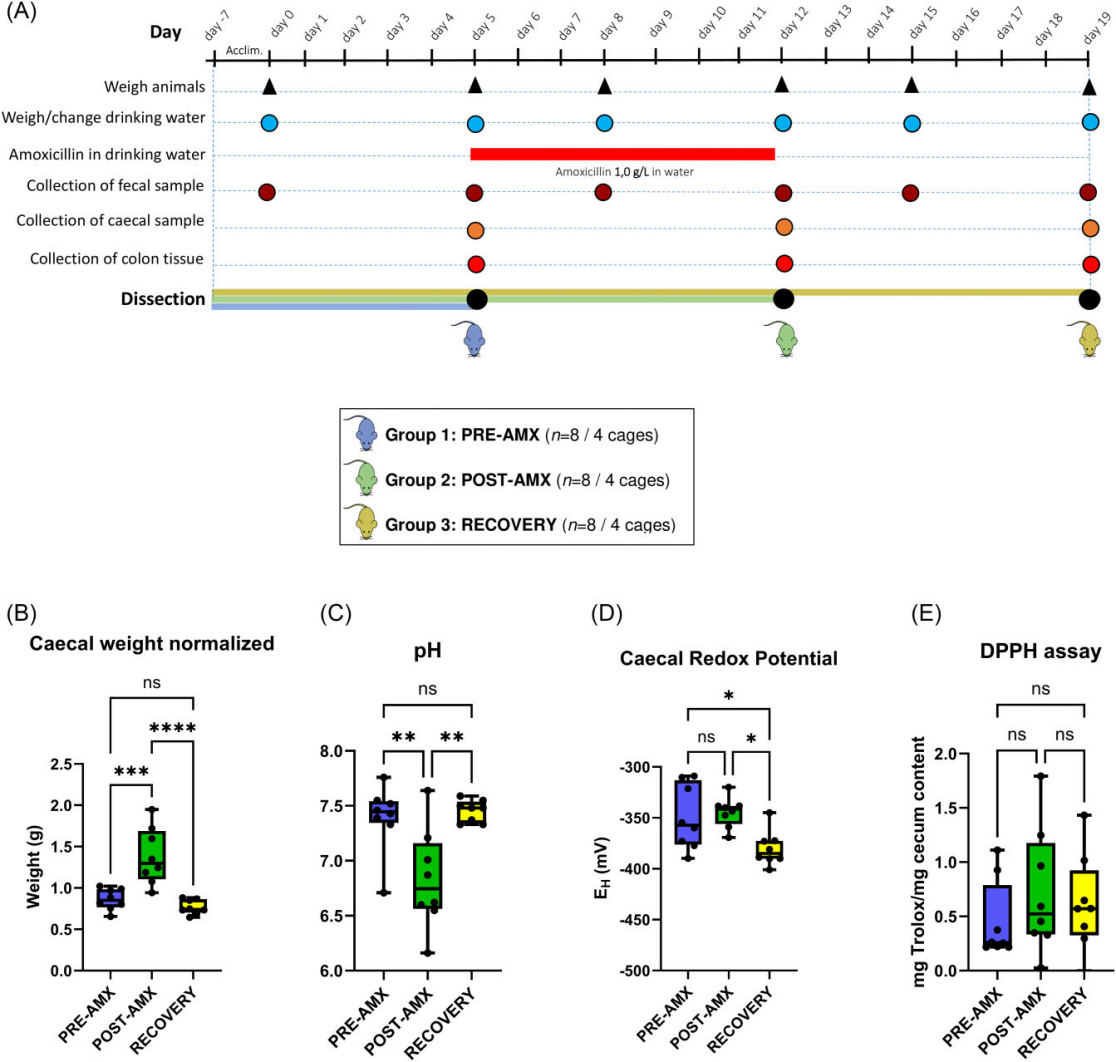
(A) Experimental design of the animal study. The animal experiment started on Day 0 after 7 days of acclimatization. After 5 days, group 1 was euthanized. On Day 5, amoxicillin (1 g/l) was given to groups 2 and 3 in the drinking water from Day 5 to Day 12, after which AMX water was removed and group 2 euthanized. Group 3 continued on normal drinking water for 7 additional days until euthanization. (B) Caecal weight, (C) pH, (D) redox potential, and (E) antioxidant capacity data obtained from the measurements of the rats’ cecum. One-way ANOVA and Tukey's multiple comparisons tests were applied to evaluate the differences between the different groups of animals at time points before, after antibiotics, and during the recovery period for (B)–(D). No significant differences were observed after the Kruskal–Wallis test was used to compare the mg of Trolox equivalents (antioxidant standard) at the different time points for (E). ns indicates no significant differences. *P < .05, **P < 0.01, ***P < 0.001.

Starting from 8 to 9 weeks of age rats were housed in 12 cages with two animals in each. After an acclimatization period of 12 days, the 24 animals were randomized by weight, and divided into three different groups (Group 1: ‘PRE-AMX’, Group 2: ‘POST-AMX’, and Group 3: ‘RECOVERY’). Amoxicillin was delivered to the animals in groups 2 and 3, dissolved in the drinking water, with a final concentration of 1 g/l for 7 days (from Day 5 to Day 12). Drinking water bottles were changed every 3–5 days. Water intake and animal body weight were monitored every 3–5 days during the whole duration of the animal experiment. A faecal sample was collected from all animals on Days 0, 5, 8, 12, 15, and 19 for archaeal and bacterial load quantification (Fig. 1). All animals in each of the three groups were euthanized on the same day by exsanguination after carbon dioxide inhalation as anaesthesia at the three different time points: On Day 5; before antibiotic treatment, Day 12; after antibiotic treatment, and Day 19; after 1 week of recovery (Fig. 1). The intestine was excised, the cecum was weighed, and its content was collected in tubes in dry ice and stored at −80°C for microbiota and metabolome analysis. The cecum was used to measure the redox potential and pH after 20 min of being excised. Proximal colon tissue samples were collected and stored at −20°C in RNA later for further RNA extraction and gene expression analysis.

### Second animal experiment

Rats of 8–9 weeks of age were housed in 10 separate cages, with two animals in each. Following a 5-day acclimatization period, animals were randomized by weight, and divided into two different groups with 10 rats in each (Group 1: ‘CTR’, and Group 2: ‘AMX’). A solution of amoxicillin at a final concentration of 30 mg/0.5 ml was delivered to the animals in group 2, by oral gavage, for 3 consecutive days (from Day 0 to Day 2). Animal body weight was monitored at Day 0 and Day 3. Faecal samples were collected every day between Day 0 and Day 3. Rats were euthanized (as described above) on Day 3 and redox potential of the cecum was measured after weighing it, and its content was collected from each rat and stored at −80°C for further analyses (Supplementary Fig. 1).

### Redox potential and pH measurements in rat's cecum

Cecum redox potential and pH measurements were carried out following an established protocol (Kimsé et al. 2009). The authors reported that *Eh* (redox potential) of the cecum in rabbits was approximately stable after 20 min *post mortem* when *in vivo* measurements were taken every 5 min up to 35 min. Considering this, and after a preliminary study, the redox potential was measured 20 min after the cecum was extracted from each rat.

Redox potential was measured *in situ*, inside the cecum by using the redox potential probe (ORP electrode InLab® Redox Micro, Mettler-Toledo). Redox potential measurements were not possible to obtain from other gut compartments. Before use, a redox measurement was performed using the calibration solution 220 mV to confirm correct functioning of the redox probe. pH measurements were obtained by the microelectrode probe (pH Sensor InLab® Ultra-Micro-ISM from Mettler-Toledo). Prior to use, the electrode was calibrated by using buffer solutions with pH 4, pH 5, and pH 9 at room temperature.

### RNA extraction and cDNA synthesis

Total RNA was extracted from approximately 20 mg of colonic tissue using the RNeasy Plus Mini Kit (Qiagen) and RNase-DNase free set (Qiagen) following the supplier's instructions. The RNA concentration and purity was determined using a Nanodrop Spectrophotometer ND-1000 (Thermo Scientific). cDNA was prepared from 600 ng RNA in 20 µl reactions using the Omniscript RT Kit (Qiagen) following manufacturing instructions.

### Gene expression analysis

The relative gene expression of the enzymes Dual oxidase-1, Dual oxidase-2, NADPH oxidase-1, NADPH oxidase-3, the tight junction proteins Claudin-1, and Occludin, the alpha subunit of the HIF-1α, as well as Mucin 2, and the enzymes catalase, superoxide dismutase, were determined by real-time reverse transcription polymerase chain reaction (RT-PCR) using TaqMan™ Universal PCR master mix and rat specific TaqMan™ Gene Expression Assays: Rn00667869_m1 Actb, Rn01775763_g1 Gapdh, Rn00596688_m1 Duox1, Rn00666512_m1 Duox2, Rn00586652_m1 Nox1, Rn01430441_m1 Nox3, Rn00580064_m1 Ocln, Rn00581740_m1 Cldn1, Rn01472831_m1 Hif1a, Rn01498206_m1 Muc2, Rn01512560_m1 Cat, and Rn00566938_m1 Sod1. Reactions were run in QuantStudio™ 6 and 7 Flex Real-Time PCR System (Applied Biosystem). Reaction conditions were as follows: Incubation at 50°C for 2 min, enzyme activation at 95°C for 20 s, followed by 40 cycles of denaturation step at 95°C for 1 s and annealing/extension at 60°C for 20 s. Results were analysed with the Quant Studio Real-Time PCR software v1.6 using the second derivative maximum method to determine CT-values using Gapdh and Actb as internal housekeeping gene controls. The delta delta CT (ΔΔCT) method was used to calculate gene expression fold change by using the mean CT-value of the PRE-AMX samples as reference to normalize values for each gene separately. The reaction followed the manufacturer's recommendations for TaqMan assay. Results were expressed as log_2_ of the fold change.


\begin{eqnarray*}
{\Delta\!\! \textrm{ CT}} = \textrm{ C}{{\textrm{T}}_{\mathrm{gene}}} - \textrm{ CT}{_{\mathrm{housekeeping}}}
\end{eqnarray*}

\begin{eqnarray*}
\Delta \Delta \textrm{CT} = \Delta \!\!\textrm{ CT}{_{\mathrm{sample}}} - \textrm{mean}\Delta \textrm{ CT}{_{\mathrm{reference}}}
\end{eqnarray*}

\begin{eqnarray*}
\textrm{Fold}\ \textrm{change}\ \left( {\textrm{FC}} \right) = {{2}^{ - \Delta \Delta \mathrm{CT}}}
\end{eqnarray*}

\begin{eqnarray*}
\log_{ 2}\!\left( {\textrm{FC}} \right) = - \Delta \Delta \textrm{CT}
\end{eqnarray*}

### *In vitro* determination of antioxidant capacity in caecal samples

The DPPH radical scavenging capacity of caecal content was performed by optimizing the method previously described (Lin and Chang 2000). Briefly, approximately 50 mg of caecal content from each rat was homogenized for 3 min at 30 cycles/s in a bead beater MM300 (Retsch, VWR, Haan, Germany) with 12 volumes of PBS. Samples were centrifuged at 15 000 *g* for 10 min at 4°C, and supernatant was obtained to mix with 0.2 mM DPPH ethanoic solution (1:60 final dilution factor). A standard curve was calculated by measuring absorbance at 517 nm with different concentrations of Trolox in methanol (0.1; 0.08; 0.04; 0.02; 0.01 mg/l). Each sample was prepared in duplicates and the %DPPH scavenging capacity was calculated after obtaining the absorbance values for samples, standards, and control (PBS) by using an EnSpire® Multimode Plate Reader (PerkinElmer). Data were expressed by mg Trolox/mg cecum content using the values obtained from the standard curve (Supplementary Fig. 2).


\begin{eqnarray*}
\% \textrm{DPPH}\ \textrm{scavenging}\ \textrm{capacity} = \ \left[ {\left( {\textrm{Abs}_{\mathrm{ c}}} - \textrm{Abs}_{\mathrm{ s}} \right)/\textrm{Abs}_{\mathrm{ c}}} \right]\ \times 100
\end{eqnarray*}

where Abs_c_ is the absorbance of the control and Abs_s_ the absorbance of each caecal sample or standard.

### DNA extraction

DNA was extracted from ∼200 mg of stool using the DNeasy® PowerLyzer® PowerSoil® DNA isolation kit (Qiagen, Germany) according to the manufacturer's instructions. Mechanical lysis of bacteria was conducted at 30 cycles/s for 10 min on a bead beater MM300 (Retsch, VWR, Haan, Germany). DNA concentrations were measured by the Qubit ds DNA BR kit (Life Technologies, Carlsbad, CA, United States), and DNA was stored at −20°C for further bacteria composition analysis.

### Archaeal primer design and validation

Universal primers and probe targeting known archaea were designed based on alignments of the 16S rRNA gene obtained from 10 selected species of archaea previously shown to inhabit the gut. The species chosen were: *Methanobrevibacter smithii, Methanosphaera stadtmanae, Methanobrevibacter oralis, Methanomassiliicoccus luminyensis, Methanocellus chikugoensis, Methanobrevibacter arboriphilus, Methanobrevibacter millerae, Haloferax alexandrinus, Halosimplex carlsbadense*, and *Halorubrum saccharovorum*. Oligo Analysis Tool from Eurofins, and the Probe Match tool from RDP (Cole et al. 2005) were used to ensure the matching efficiency. *In vitro* testing was performed using the DNA sequences from the same archaea used for *in silico* analysis; *M. smithii* (DSM 861), *M. stadmanae* (DSM 3091), *M. chikugoensis* (DSM 13459), *H alexandrinus* (DSM 27206), and *M. luminyensis* (DSM 25720). The following primers were selected: Arc_fwd_4 AAACTTAAAGGAATTGGCGGGG and Arc_rev GGGTCTCGCTCGTTGCC based on the highest coverage (78.1% and 96.1%) with 0 and 1 mismatch, respectively (Supplementary Fig. 3). *In vitro* testing by PCR with AccuPrime™ *Taq* DNA Polymerase (0.8 µl) and 10 µM of each of the selected primers, followed by gel electrophoresis (E-Gel^®^ 1.2% with SYBR Safe, Invitrogen) showed amplification of all the expected species except *M. luminyensis*. Reaction conditions were as follows: pre-incubation at 94°C for 2 min followed by 35 cycles of 94°C for 20 s, 54°C for 20 s, and 68°C for 30 s. Lastly, a cycle at 68°C for 5 min before cooling at 4°C. As a negative control, *Faecalibacterium prausnitzii* (DSM 17677) was used (data no shown).

### Archaeal quantification by qPCR

DNA samples from caecal content were diluted to 5 ng/µl. Amplifications were carried out in triplicate in a 384-well format using a LightCycler^®^ 480 II (Roche Applied Science) and analysed using the LightCycler^®^ 480 Software. To quantify archaea by qPCR, a fragment of the 16S rRNA gene was amplified by using the selected primer pair: Arc_fwd_4 AAACTTAAAGGAATTGGCGGGG and Arc_rev GGGTCTCGCTCGTTGCC. Each qPCR reaction contained 5 µl of SYBR Green I Master (Roche Applied Science, Penzberg, Germany) 0.5 µM of each primer, and 2 µl of diluted template DNA (1 ng/µl) in a total reaction volume of 10 µl. Reaction conditions were: pre-incubation at 95°C for 5 min followed by 45 cycles of 95°C for 10 s, 57°C for 15 s, and 72°C for 15 s. Lastly, a melting curve was generated (95°C for 5 s, 68°C for 1 min, and increasing the temperature to 98°C with a rate of 0.11°C/s with continuous fluorescence detection). Ten-fold dilution series of purified *M. smithii* DNA (DSM 861/ATCC 35061) were used for quantification of the 16S rRNA gene in archaea by establishing standard curves (16S rRNA gene copies/2 µl vs. CT values) and performing linear regression. Based on CT values, the corresponding number of 16S rRNA genes counts were calculated per mg caecal content:


\begin{eqnarray*}
\textrm{archaeal}\ \textrm{load}\ /\ \textrm{mg}\ \textrm{of}\ \textrm{caecal}\ \textrm{content}\ = \frac{{\left( {\textrm{number}\ \textrm{of}\ 16\textrm{S}\ \textrm{rRNA}\ \textrm{gene}\ \textrm{copies}/2\,\mu \textrm{l}} \right)\times \textrm{dilution}\ \textrm{factor}\ \textrm{for}\ \textrm{the}\ \mathrm{DNA\, cc}\times \textrm{DNA}\ \textrm{extraction}\ \textrm{elution}\ \textrm{volume}\ of\ 80\,\mu \textrm{l}}}{{\textrm{mg}\ \textrm{of}\ \textrm{caecal}\ \textrm{content}}}
\end{eqnarray*}

### Bacterial load

Bacterial load was estimated by quantification of the copy number of 16S rRNA gene by qPCR as previously described (Tulstrup et al. 2015). Briefly, a fragment of the V3-region of the 16S rRNA gene was amplified in triplicate for each sample, using universal primers: PBU CCTACGGGAGGCAGCAG and PBR ATTACCGCGGCTGCTGG. Each qPCR reaction contained 5 µl SYBR Green I Master (Roche Applied Science, Penzberg, Germany), 0.5 µM of each primer (10 µM), and 2 µl of diluted template DNA (1 ng/µl) in a total reaction volume of 10 µl that was reached with 2 µl PCR-grade water. Reaction conditions were as follows: pre-incubation at 95°C for 5 min followed by 45 cycles of 95°C for 10 s, 55°C for 15 s, and 72°C for 15 s. Lastly, a melting curve was generated (95°C for 5 s, 68°C for 1 min, and increasing the temperature to 98°C with a rate of 0.11°C/s with continuous fluorescence detection). The qPCR was run in 384-well format on a LightCycler^®^ 480 II (Roche Applied Science) and analysed using the LightCycler^®^ 480 software. Tenfold dilutions of purified *E. coli* MG1655 (DSM 18039) DNA were used for quantification of the 16S rRNA gene in bacteria by establishing standard curves (16S rRNA gene copies/2 µl vs. CT values) and performing linear regression. Based on CT values, the corresponding number of 16S rRNA genes counts were calculated per mg caecal content:


\begin{eqnarray*}
\textrm{bacterial}\ \textrm{load}\ /\ \textrm{mg}\ \textrm{of}\ \textrm{caecal}\ \textrm{content}\ = \frac{{\left( {\textrm{number}\ \textrm{of}\ 16\textrm{S}\ \textrm{rRNA}\ \textrm{gene}/2\,\mu \textrm{l}} \right)\times \textrm{dilution}\ \textrm{factor}\ \textrm{for}\ \textrm{the}\ \textrm{DNA}\ \textrm{cc}\times \textrm{DNA}\ \textrm{extration}\ \textrm{elution}\ \textrm{volume}\ \textrm{of}\ 80\,\mu \textrm{l}}}{{\textrm{mg}\ \textrm{of}\ \textrm{caecal}\ \textrm{content}}}
\end{eqnarray*}

### 16S rRNA amplicon sequencing

DNA extracted from the caecal samples was used for sequencing the V3-region of the 16S rRNA gene to analyse the bacterial community composition, as previously described (Christensen et al. 2014). Briefly, the V3-region of the 16S rRNA gene was amplified using a universal forward primer (PBU 5′-A-adapter-TCAG-barcode-CCTACGGGAGGCAGCAG-3′) with an unique 10–12 bp barcode for each sample (Ion Xpress barcode as suggested by the supplier, Life Technologies) and a universal reverse primer (PBR 5′-trP1-adapter-ATTACCGCGGCTGCTGG-3′). The PCR reactions were conducted with 4 µl HF-buffer, 0.4 µl dNTP (10 mM of each base), 2 µM forward primer, 2 µM reverse primer, 5 ng template DNA in 1 µl, and 0.2 µl Phusion High-Fidelity DNA polymerase (Thermo Fisher Scientific, Waltham, MA, United States) in a total reaction volume of 20 µl. Reaction conditions were as follows: initial 98°C for 30 s followed by 24 cycles of 98°C for 15 s and 72°C for 30 s, and finally, 72°C for 5 min before cooling to 4°C. PCR products were purified by HighPrepTM PCR Clean-up System (Magbio, Gaithersburg, MD, United States) according to the manufacturer's protocol, and DNA concentrations were determined with Qubit HS assay. Finally, a library was constructed by mixing an equal amount of PCR products from each sample. Sequencing was performed on the Ion OneTouch^TM^ 2/Ion S5^TM^ sequencing system with an Ion 520/530™ Kit 520-chip (ThermoFischer Scientific). Sequence data are available at the NCBI's Sequence Read Archive under BioProject number PRJNA1054039.

### Sequence data handling

Sequence data were obtained in FASTQ format and further processed using CLC BIO genomic workbench (Qiagen Hilden, Germany) to de-multiplex and remove sequencing primers, filtered reads with a final length between 125 and 180 bp were exported in FASTQ format. Further quality trimming using default settings (remove low-quality nucleotides base-calling error = 0.05, trim ambiguous nucleotides = 2) was performed in DADA2 (Version 1.12.2) with default settings as described previously (Callahan et al. 2016). Finally, an amplicon sequencing variant (ASV) table was constructed which contains the counts of each sequence variant in each sample. All sequence reads were taxonomically classified using the Ribosomal Database Project Multi-classifier tool (Wang et al. 2007) against the RDP database v18.

The ASV table was imported into the Qiime2 pipeline (Version 2021.11), and α and β diversity metrics were calculated by the function ‘diversity core-metrics-phylogenetic’ based on a rooted phylogenetic tree (Bolyen et al. 2019). For analysis of faecal samples only, samples were rarefied to 17 000 reads to eliminate bias from uneven sampling depth.

### SCFAs and organic-amino acid analyses

Caecal samples were prepared for short-chain fatty acids (SCFAs), amino acids, and organic acids analysis by GC–MS. For the sample extraction, between 100 and 150 mg of cecum content was weighed in a 1.5 ml Eppendorf tube. Caecal samples and extracts were kept on ice or at 4°C throughout sample preparation. A volume of 4x wt/vol MilliQ® water was added to each sample. Samples were vigorously vortexed for 1 min until the suspension was reached. Samples were at centrifuged at 15 000 *g* for 10 min at 4°C and the supernatant was transferred into a labelled SpinX (Costar) centrifuge filter. Samples were filtered by centrifugation at 15 000 *g* for 5 min at 4°C. The extracted caecal water was stored at −80°C until shipment to MS-Omics for its analysis. Sample analysis was carried out by MS-Omics as follows. Samples for SCFAs analysis were acidified using hydrochloride acid and deuterium-labelled internal standards were added. Analysis was performed using a high polarity column (Zebron™ ZB-FFAP, GC Cap. Column 30 m × 0.25 mm × 0.25 µm) installed in a GC (7890B, Agilent) coupled with a quadrupole detector (5977B, Agilent). Samples for amino acids and organic acids were derivatized with methyl chloroformate using a slightly modified version of the protocol described by Smart et al. (2010). Analysis was performed using gas chromatography (7890B, Agilent) coupled with a quadrupole mass spectrometry detector (5977B, Agilent). In both analyses, the system was controlled by ChemStation (Agilent). Raw data was converted to netCDF format using Chemstation (Agilent) before the data were imported and processed in Matlab R2018b (Mathworks, Inc.) using the PARADISe software (Johnsen et al. 2017). All samples were analysed in a randomized order.

### Statistical analysis

Differences between beta-diversity of groups were assessed by applying ANalysis Of SImilarities ANOSIM (CLARKE 1993) to weighted and unweighted UniFrac distances in Qiime 2. Graphs and additionally statistically analyses were generated in Prism version 9.1.1 (GraphPad, San Diego, CA, USA) and JMP Pro 14 (SAS, Durham, NC, USA). To minimize false discoveries in univariate statistics, ASVs not present in at least 50% of the samples of at least one of the compared groups were considered rare and excluded from the analysis. Filtered taxa differences between groups, alpha-diversity indices (Shannon and Observed ASV), abiotic factors (pH, redox potential, caecal weight, antioxidant capacity) comparisons between groups, together with qPCR data, and SCFAs were analysed by one-way ANOVA when data passed Shapiro–Wilk normality test, and Kruskal–Wallis when not normally distributed or variances were significantly different, followed by Dunn's or Tukey's multiple comparisons test. SCFAs and organic amino acids multivariate analyses were performed in R (v.4.2) (R Core Team 2022) using the package FactoMineR and function PCA (Husson 2010). The heatmap at genus level was generated with the Pheatmap package (Kolde R 2019). *P*-values were adjusted using the Benjamini–Hochberg step-up method with a false discovery rate (FDR) of 0.05. For all statistical analyses, significance was set at *P* < .05 (Benjamini et al. 2006).

## Results

### Oral antibiotic treatment acutely affects the caecal pH and size, but not redox potential or antioxidant capacity in Wistar Han rats

To test our hypothesis we conducted an animal experiment with Wistar Han rats exposing them to amoxicillin via their drinking water (Fig. 1A). Water consumption was not significantly affected by the addition of amoxicillin (Kruskal–Wallis test, Supplementary Fig. 4). A greater mean weight of the cecum excised from the POST-AMX rats was observed compared to those of the PRE-AMX rats (*P* < .001, Fig. 1B) and the RECOVERY rats (*P* < .0001, Fig. 1B), while no difference was observed between PRE-AMX and RECOVERY rats (*P* > .05, Fig. 1B). A lower mean pH in the cecum of the POST-AMX rats was observed, compared to the mean pH in the PRE-AMX (*P* = .006, Fig. 1C), and in the RECOVERY rats (*P* = .003, Fig. 1C), while no difference was measured between PRE-AMX and RECOVERY rats (*P* > .05, Fig. 1C). No significant differences were seen in the mean redox potential in the cecum of the POST-AMX rats compared to PRE-AMX rats (*P* = 0.899, Fig. 1D), however, a significantly lower redox potential was observed in the cecum of the RECOVERY rats compared to both the PRE-AMX (*P* = .032, Fig 1D) and the POST-AMX (*P* = .012, Fig. 1D). When we assessed the antioxidant capacity of cecum content using the DPPH assay we did not find any significant differences with the Kruskal–Wallis test between PRE-AMX, POST-AMX, and RECOVERY groups (Fig. 1E). As our primary aim was to study the acute effects of antibiotics on redox-potential and antioxidant capacity, but the cecum samples were not assessed on the same day for both amoxicillin and control groups, we performed an additional animal study with same age Wistar Han rats to confirm our findings. For this study we included 10 animals as a control group, and 10 animals that received amoxicillin (amoxicillin group) by oral gavage during 3 days. Animals from both groups were euthanized on the same day (Day 3) and redox potential was measured inside the cecum. However, this experiment confirmed the finding from our first animal experiment, as no differences were observed in caecal antioxidant capacity or caecal redox potential between CTR and AMX. Consistent with the first experiment, ceca were larger in the AMX than in the CTR group (*P* < .001) (Supplementary Fig. 1).

### Amoxicillin treatment disturbs the intestinal microbiome showing non-complete recovery 1 week after cessation of treatment

**Figure 2.**
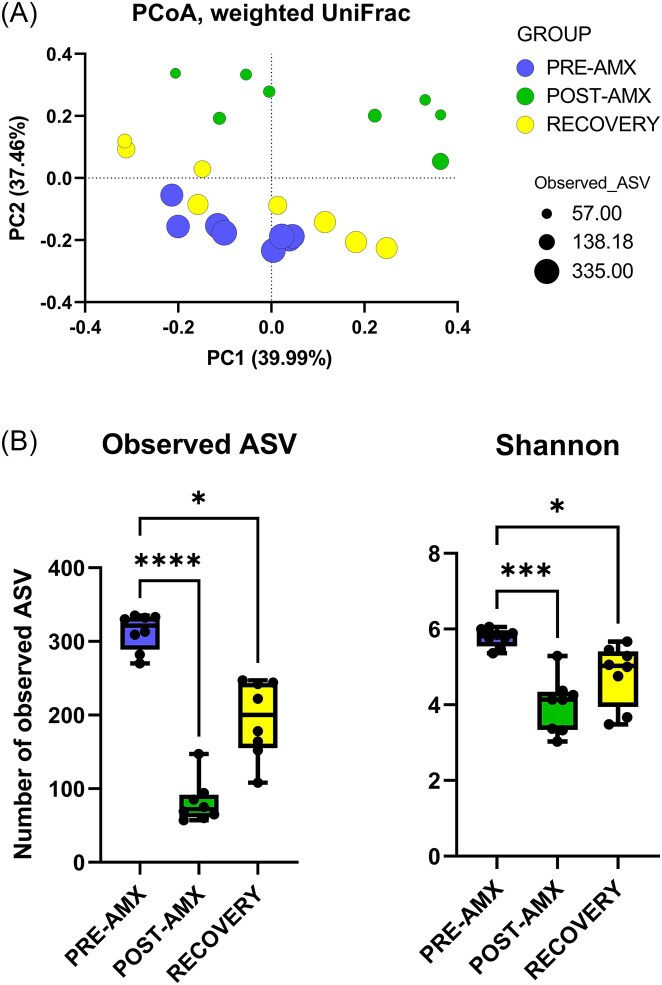
(A) 2D PCoA plot based on weighted UniFrac distances of the microbiome in caecal samples. Significant differences were observed by ANOSIM when both PRE-AMX vs. POST-AMX (weighted UniFrac ANOSIM: *R* = 0.902; *P* = .002), and POST-AMX vs. RECOVERY (weighted UniFrac ANOSIM: *R* = 0.633; *P* = 0.002) were compared. After 1 week of recovery, the caecal microbiome composition was still significantly different from the baseline (weighted UniFrac ANOSIM: *R* = 0.445; *P* = .003). (B) Observed ASV and Shannon index as metrics to measure the alpha diversity in the cecum of the animals. One-way ANOVA and Tukey's tests for Shannon and non-parametric Kruskal–Wallis and Dunn's tests for Observed ASVs were used to compare the differences in alpha diversity between groups of animals.

Principal coordinate analysis (PCoA) of the weighted UniFrac distance matrix revealed visible clustering of caecal microbiota reflecting the three groups. This was confirmed by ANOSIM analysis, which showed significant differences between the groups PRE-AMX vs. POST-AMX (*R* = 0.902; *P* = .002), and the groups POST-AMX vs. RECOVERY (*R* = 0.633; *P* = .002). Although getting closer to the cluster of the PRE-AMX group, the RECOVERY group was still significantly different from this group (*R* = 0.445; *P* = 0.003) (Fig. 2A).

As expected, caecal microbiota alpha diversity was significantly lower in the POST-AMX than in the PRE-AMX group for both Observed ASVs (*P* < .001) and Shannon index (*P* < .001). One week after cessation of antibiotic use (RECOVERY), the caecal microbiome was characterized by a higher observed ASV and Shannon index, becoming more similar to PRE-AMX. However, 1 week was not sufficient to revert to the initial alpha diversity. These data confirm that amoxicillin delivery in the drinking water affects the composition of the cecum microbiota in healthy Wistar Han rats, and that the microbiome requires more than 1 week of recovery to reach the microbiome composition and diversity observed before antibiotic treatment (Fig. 2B and Supplementary Fig. 5).

**Figure 3.**
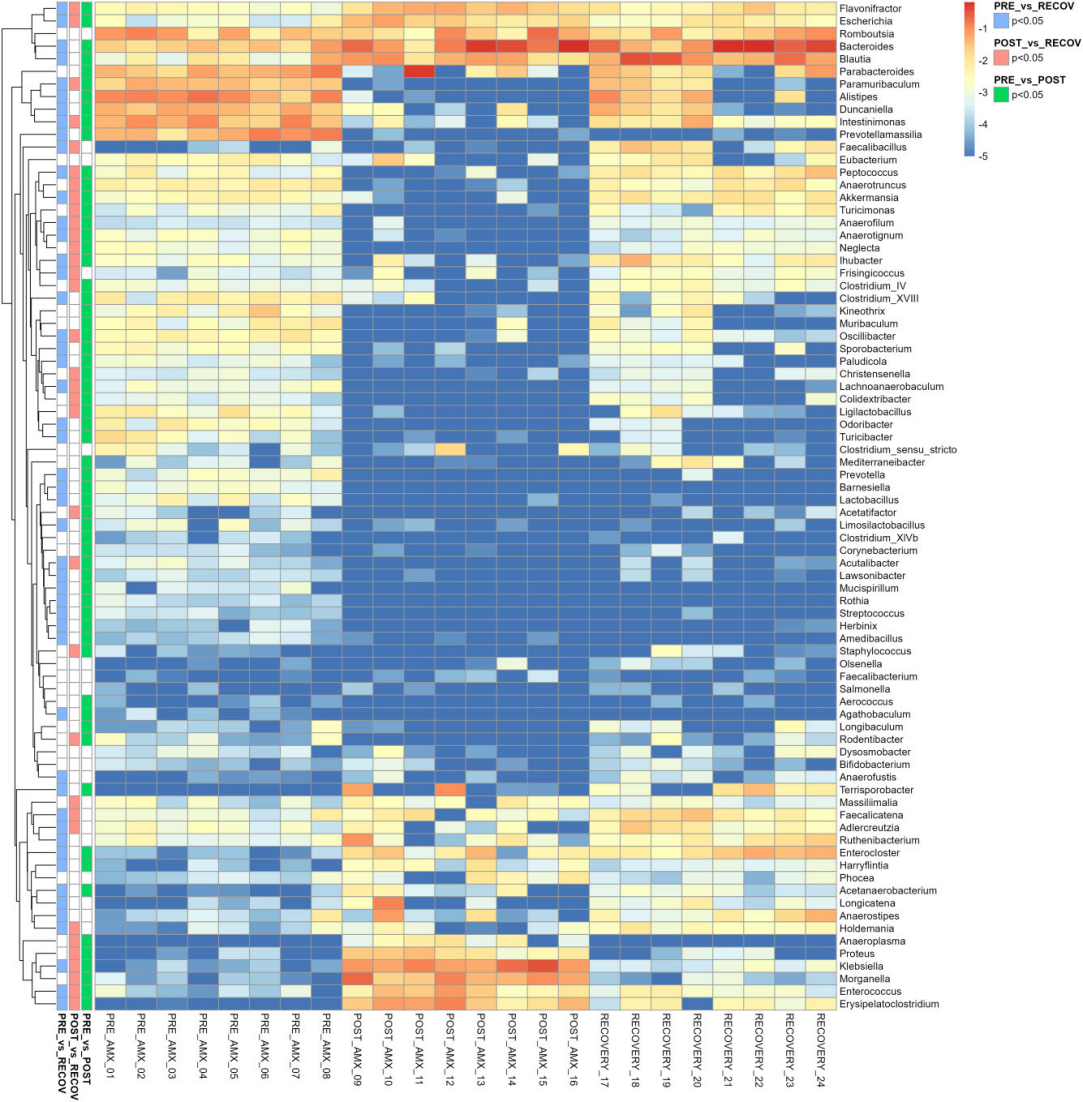
Heatmap based on the log of the relative abundance at the genus level for the different time points in the caecal samples of the animals. The order of the genera is based on Euclidean clustering. Significant differences between groups as defined by the FDR applied to the *P* values (<.05) are specified for the different comparisons on the left side of the heatmap.

**Figure 4.**
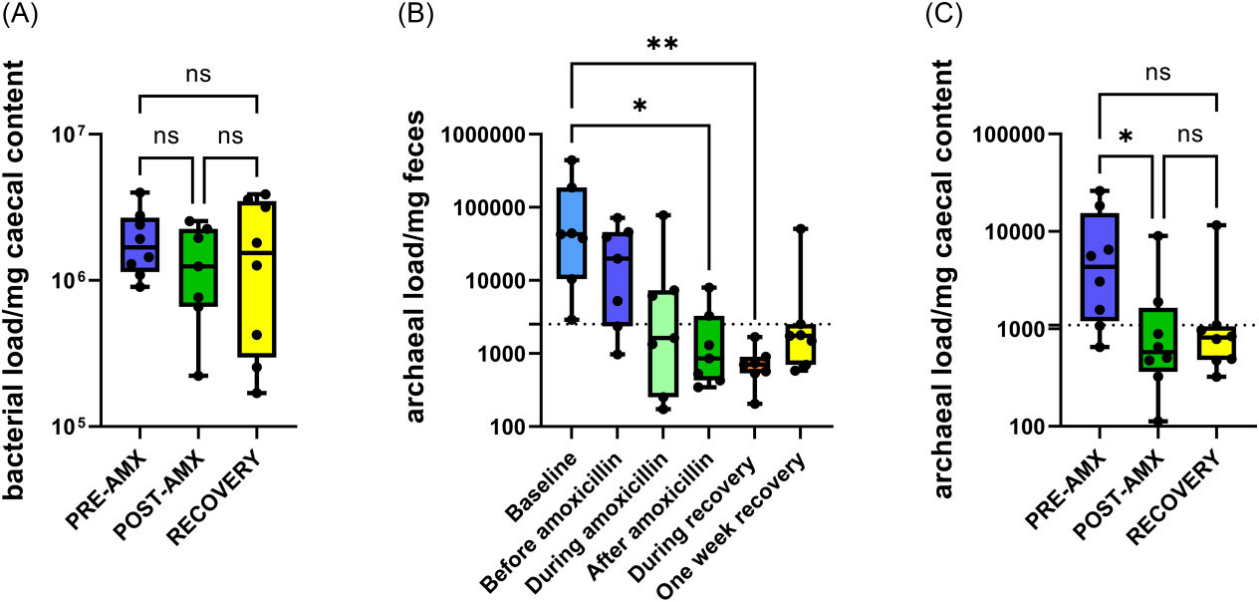
(A) Bacterial load per mg of rat's caecal content. Quantitative PCR results show no significant differences before, after amoxicillin, or during recovery. Kruskal–Wallis and Dunn's tests were used to compare the different time points. (B) Archaea copy number per mg of rat's faeces over time. Quantitative PCR results show an overall decrease in the number of copies of archaeal DNA in the faecal samples over the time of the animal experiment. Friedman and Dunn's tests were performed to compare between the different time points. (C) Archaea copy number per mg of rat's cecum in the different groups. Kruskal–Wallis and Dunn's tests show a significant decrease in the archaeal copy number per mg of caecal content after antibiotic treatment, which is not recovered after a week of having finished the antibiotic treatment. Grid line indicate LOD/2.

A decreased relative abundance of most of the genera was observed in the POST-AMX vs. PRE-AMX group (Fig 3). Some of them recovered after one week, however, most of the strict anaerobes were completely depleted such as *Prevotella, Prevotellamassilia* (Takeuchi et al. 2000), and did not reach their original abundances in the RECOVERY group when compared to PRE-AMX. Nevertheless, not only the strict anaerobes were affected by the antibiotic treatment, also some genera known as aerotolerant or facultative anaerobes such as *Rothia* (West et al. 2024)*, Lactobacillus* (Falsen et al. 1999), and *Limosilactobacillus* (Saroha et al. 2023) were present in lower abundances after one week of recovery (Fig. 3). Interestingly, the strict anaerobe, *Blautia* (Park et al. 2012), and *Bacteroides* (Patrick 2002), increased in relative abundance POST-AMX and also during recovery (Fig. 3). Facultative anaerobes such as *Enterococcus*, and most of the genera within the Gammaproteobacteria class: *Escherichia/Shigella, Klebsiella, Morganella*, and *Proteus*, among others (Bruckner et al. 1999, Moreira de Gouveia et al. 2024) were present at higher relative abundances after antibiotic treatment, which consistently decreased after the recovery time (Fig. 3). The average total bacterial load did not change during the study (Fig. 4A). Archaeal load in faeces of the RECOVERY group decreased during (*P* = 0.005) and shortly after amoxicillin treatment (*P* = 0.025) and did not fully recover after amoxicillin treatment ceased (Fig. 4B). Archaeal load in caecal samples showed a significantly lower count in the POST-AMX group when compared to the PRE-AMX group (*P* = .006) but did not differ significantly when compared to the RECOVERY group (Fig. 4C), although it was at a comparable level.

### Amoxicillin treatment affects caecal microbial-derived metabolites being fully recovered 1 week after cessation of treatment

**Figure 5.**
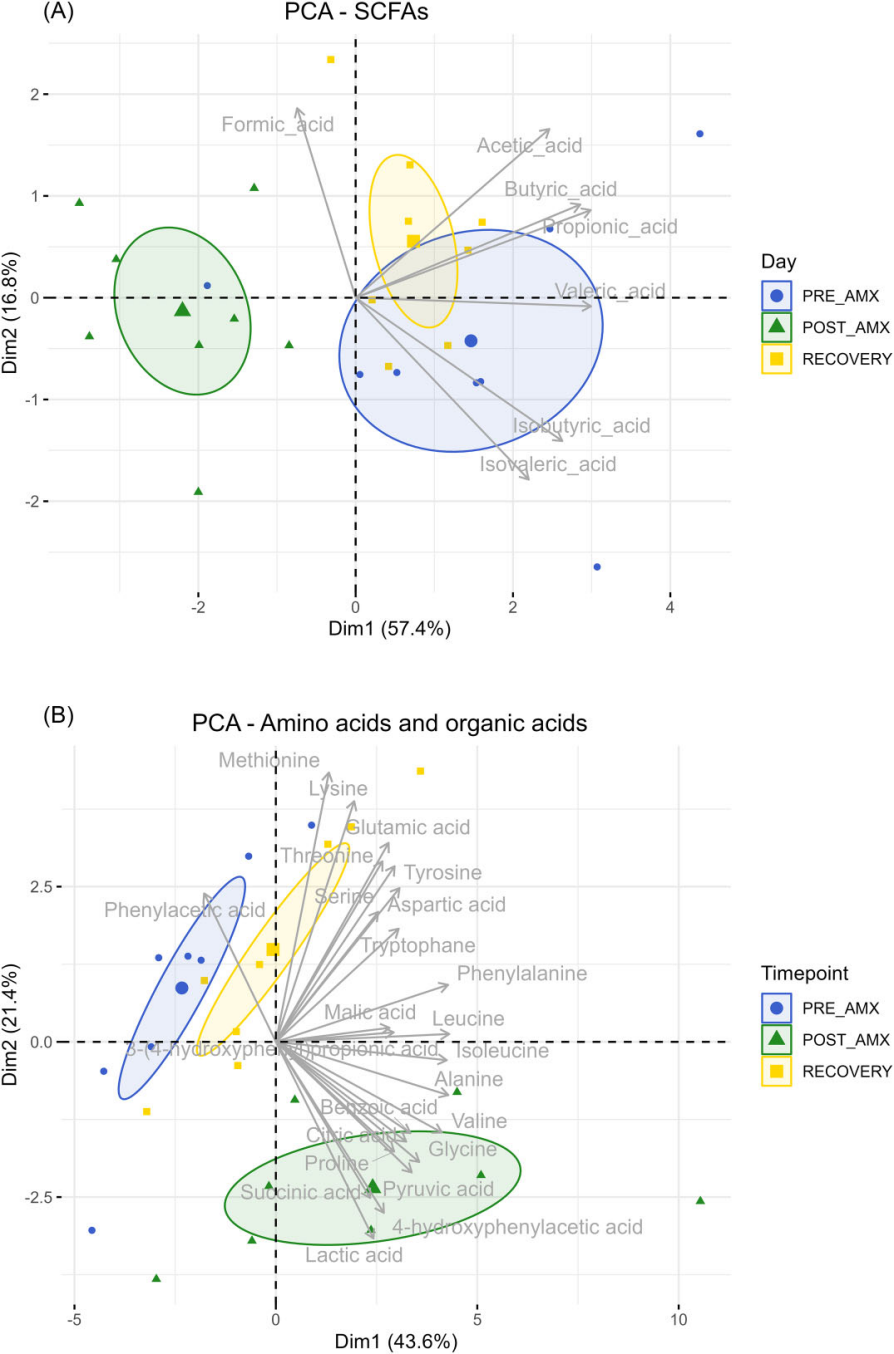
(A) 2D PCA plot of the SCFAs in caecal samples. Significant differences were observed in the PCA dimension one by ANOVA followed by Tukey test when both PRE-AMX vs. POST-AMX (*P* < .001) and POST-AMX vs. RECOVERY (*P* < .001) were compared. After 1 week of recovery, the overall caecal SCFAs concentration was recovered, being not significantly different from the baseline values (*P* = .516). (B) 2D PCA plot of the organic acids and amino acids in caecal samples. Significant differences were observed in the PCA dimension two by ANOVA and Tukey tests when both PRE-AMX vs. POST-AMX (*P* = .004) and POST-AMX vs. RECOVERY (*P* < .001) were compared. After one week of recovery, the overall caecal organic and amino acid concentration was also recovered (*P* = .764). For dimension one a significant difference was detected for PRE-AMX vs. POST-AMX (*P* = .011), but not for the other comparisons. Ellipses show 95% confidence interval.

The PCA plot showed that amoxicillin treatment strongly affected the SCFA profiles (PRE-AMX vs. POST-AMX; *P* < .001), but they largely recovered 1 week after treatment ceased (*P* = .516) (Fig. 5A). All the individual SCFAs detected were present in lower concentrations in caecal samples in the animals of the POST-AMX group, compared to the PRE-AMX. However, the concentrations of all the individual SCFAs (except formic acid, which was not different at any time point) fully recover one week of after amoxicillin treatment, as none of them were significantly different between RECOVERY and PRE-AMX groups (Supplementary Fig. 6). Even though the gut microbiome was still affected after one week of recovery from amoxicillin treatment, the SCFAs were not, suggesting a faster recovery of some of the bacterial community functions and metabolic activities than in their composition or abundances.

Similar to the SCFAs, an almost complete recovery of the concentration of amino acids and organic acids was observed 1 week after cessation of antibiotic treatment (Fig. 5B). However, the majority of the amino acids were not affected by the antibiotic treatment. Only glycine, valine, and isoleucine increased significantly in the cecum after amoxicillin, and values returned to the baseline after the recovery time (Supplementary Fig. 7). As observed in previous studies, succinic acid was present in larger amounts after antibiotic treatment. We also observed a higher concentration of pyruvic acid, succinic acid, lactic acid, malic acid, and citric acid, which could explain the lower pH in the cecum after antibiotic treatment (Supplementary Fig. 8).

### mRNA analysis showed an increase of the scavenging enzyme catalase in the colon after amoxicillin treatment

**Figure 6.**
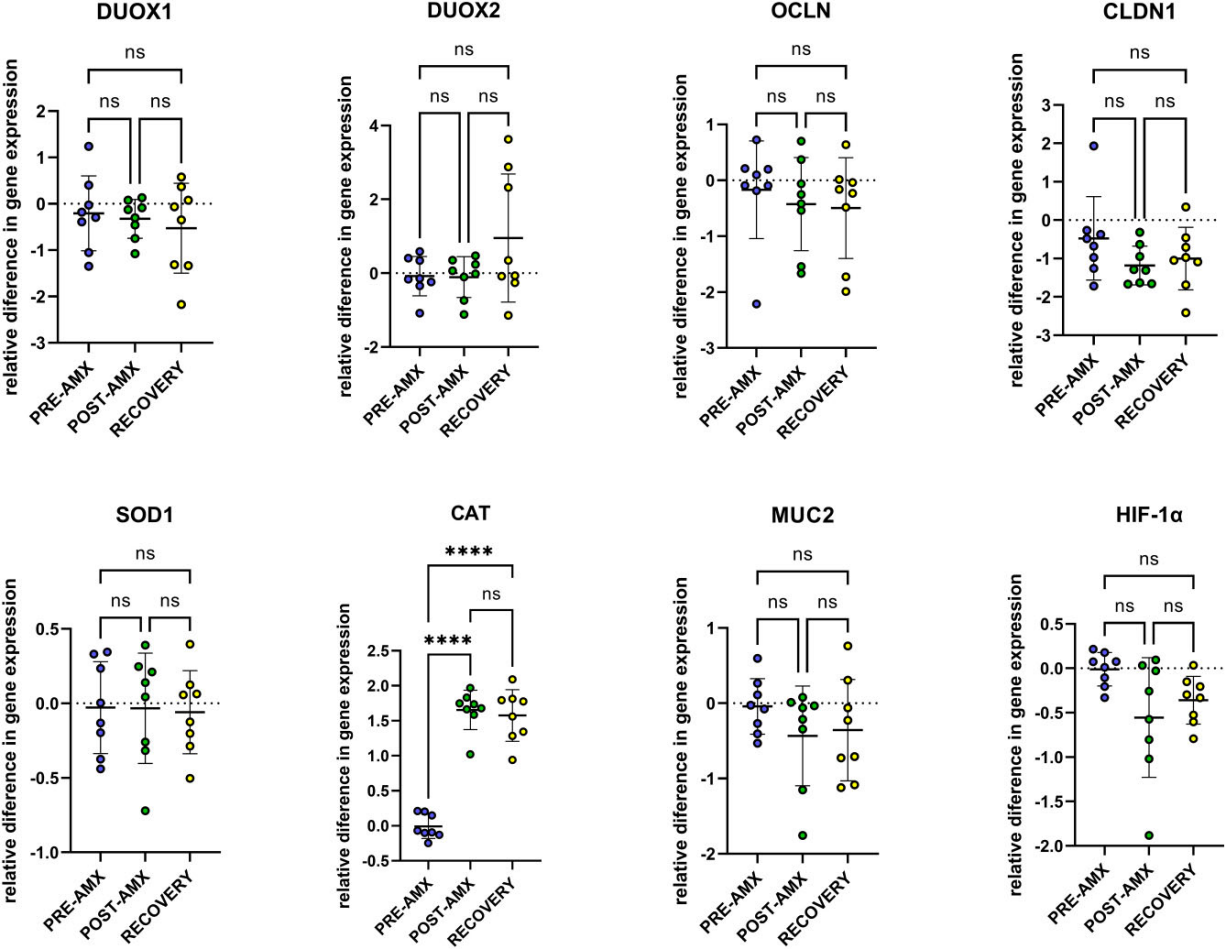
Gene expression analysis in colon tissue for all groups. Relative difference in gene expression plots is shown for those genes that had a significantly different mean when compared between groups. Log 2 of the values was used for plotting the results. One-way ANOVA and Tukey's multiple comparisons tests were applied. ns indicates no significant differences. *P < .05, **P < .01, ***P < .001, ^****^P < .0001.

We evaluated the relative expression of the genes: Dual oxidase-1 (DUOX1), Dual oxidase-2 (DUOX2), NADPH oxidase-1 (NOX1), NADPH oxidase-3 (NOX3), Occludin (OCLN), Claudin-1 (CLDN1), superoxide dismutase (SOD1), Catalase (CAT), Mucin-2 (MUC2), and HIF-1α. Results from TaqMan assay by qPCR showed only significant differences in colonic catalase expression (Fig. 6). Relative difference in expression of CAT was greater POST-AMX vs. PRE-AMX, (*P* < .001), as well as RECOVERY vs. PRE-AMX (*P* < .001). NOX1 was only detected in 5 out of 24 samples, and NOX3 in none of them. No significant differences were observed in the rest of the genes assessed (Fig. 6).

## Discussion

In this study, the redox potential measurements in the PRE-AMX treatment group did not show consistent differences compared to POST-AMX and RECOVERY groups. Although redox potential measurements obtained after oral amoxicillin showed greater values than those from rats after one week of recovery from the antibiotic treatment, the redox potential range at all time points was still within the range of what is expected from an anaerobic environment (−400 to 200 mV) (Pepper and Gentry 2015). A redox potential lower than −100 mV indicates an anaerobic environment and values greater than 100 mV indicate aerobic activity (Scholz 2019). During the experiment, when the electrode was inserted in the cecum of the rats to measure the redox potential, it decreased approximately −100 to −200 mV until stabilization of the measurement (−300 to −400 mV). This observation is in agreement with what is previously reported for pigs (Lizardo et al. 2012). A limitation of the present study may be that redox potential measurements were not performed inside an anaerobic chamber as done in a few previous studies (Friedman et al. 2017, Rongying et al. 2022). However, our data suggest that oxygen influx into the cecum has not affected our conclusions, since all our measurements are highly negative. Opposite to what is reported by Reese et al. (2018), we did not observe an increase in the redox potential after antibiotic treatment. The different outcomes could be explained by different measurement sites; we measured inside the caecum, while Reese et al. (2018) performed the measurements in faecal samples. We were limited to measure redox potential to the caecum, due to the size of the electrode probe, and the low amounts of content in the other compartments of the gut. Another difference is that we used amoxicillin, while Reese et al. (2018) used a cocktail of antibiotics, perhaps enforcing a larger and broader effect on the gut microbiota and luminal redox state. Indeed, as we observed no significant changes in bacterial load, it is plausible that a reduction in bacterial load is crucial to discern substantial changes in redox potential. We also tried to measure ROS/RNS concentration in waster cecum content with an *in vitro* ROS/RNS Assay Kit, however, the measurements were highly variable between triplicates and repeated experiments and we therefore did not trust the results obtained (data not shown). To confirm our redox potential results, an additional short term animal study was performed, using a 3-day oral delivery of amoxicillin (compared to vehicle), measuring caecal redox potential and performing DPPH assay on caecal contents, which supported our previous finding of no acute effects of this antibiotic on redox potential in the gut.

An increase in the cecum weight and size is one of the effects commonly observed after antibiotic intake (Ge et al. 2017, Zarrinpar et al. 2018). Consistently, our data from both animal experiments demonstrated enlarged caecal size. Furthermore, in our studies, the caecal luminal pH became more acidic after antibiotic treatment, which recovered after one week of the cessation of the antibiotic. Interestingly, our results show the opposite of what was found in previous rodent studies (Hentges et al. 1990, Shimizu et al. 2021), which are reported to get more alkaline gut contents after antibiotic treatment. However, this may be affected by the diet, since most standard chows are fibre rich diets that will lead to low caecal and colonic pH due to high SCFA production. This fibre fermentation may become disrupted during antibiotic treatment and lead to higher pH. However, in the present study we used a fibre-depleted diet which is also reflected in the neutral caecal pH in the antibiotics free rats. It might however also depend on the type of antibiotics as we have previously shown differential caecal pH values depending on the type of antibiotics administered to rats (Tulstrup et al. 2015, 2018).

We found a lower concentration of caecal butyrate after antibiotic treatment when compared to baseline. This is reported in previous studies after antibiotic treatment in mice and rats (Kelly et al. 2015, Smith et al. 2013). One of the explanations relies on the antibiotic action on butyrate producer members such as *Lachnospiraceae* and *Ruminococcaceae* (Marius et al. 2017), which were depleted following amoxicillin treatment (Supplementary Fig. 9). Interestingly, while *Lachnospiraceae* abundance recovered after 7 days, *Ruminococcaceae* did not (Supplementary Fig. 9). These bacteria play a crucial role in maintaining low oxygenation levels in the gut lumen through the utilization of butyrate by colonocytes and the stabilization of HIF-1α (Donohoe et al. 2012, Rivera-Chávez et al. 2016).

Previous studies report that inflammation increase the production of ROS by host immune cells through NADPH oxidase activation (Kruidenier et al. 2003, Winter et al. 2010, 2013). Consequently, bacteria with protective mechanisms against ROS, such as *Enterobacteriaceae* (Spees et al. 2013), are more likely to survive in a more oxidizing gut environment. Several studies confirm an increased *Enterobacteriaceae* family abundance after amoxicillin (Elvers et al. 2020). We saw an increase in the abundance of several members of the *Enterobacteriaceae* family, including *Escherichia*/*Shigella, Klebsiella*, and *Morganella*, known to have pro-inflammatory properties (Pittayanon et al. 2019). However, our results indicate that the expansion of these taxa is independent of luminal redox potential. Despite the greater relative abundance of these potentially pro-inflammatory bacteria, gene expression analysis did not show greater expression of NADPH oxidases, responsible among other sources, for ROS production, nor the markers of gut barrier integrity such as HIF-1α, Mucin-2, or tight junctions. However, we observed a highly significant increase in Catalase gene expression in the colon of the rats POST-AMX extending also to the recovery phase, which is known to be overexpressed when an inflammatory process is present in the intestine (Gagnière and Bonnet 2017). Distinguishing between oxygen levels, concentrations of ROS, and redox potential is crucial, as ROS generation can occur even in environments with low oxygen tensions. The lumen of the colon is anoxic, and some commensal bacteria have the ability to produce ROS to exert antimicrobial activity (Campbell and Colgan 2019). In addition, CAT catalyse the dismutation of hydrogen peroxide (H_2_O_2_) into water, generating molecular oxygen (Scandalios 2002). Low levels of CAT expression correlate with a high production of ROS (Glorieux et al. 2015), and oral administration of catalase producing *Lactoccosus lactis* is beneficial in the context of chemically induced colon cancer and inflammation in mice (De Moreno De LeBlanc et al. 2008). However, CAT regulation depends on multiple factors (Kodydková et al. 2014), and several diseases are associated with a lower expression of CAT or catalase activity, which might indicate that a correct regulation of catalase is crucial to keep the ROS homeostasis and tissue protection (Piecuch et al. 2020). Further studies are required to fully understand the implications of amoxicillin induced CAT expression. A decrease in the copy number of archaea (methanogens) in the faeces of the rats during amoxicillin treatment was observed. This is an important finding, since methanogenesis in the gut benefits the host and microbiota by enhancing secondary metabolism (SCFAs) and promoting bacterial growth through lowering hydrogen levels. In addition, archaea are not susceptible to the antibiotic-killing effect of amoxicillin, since the archaeal domain lacks peptidoglycan, the inhibition target of beta-lactam drugs. Archaea are strictly anaerobic microorganisms that are critically susceptible to redox potential and high oxygen levels, and methanogenesis depends strictly on the redox and oxygen conditions (Hirano et al. 2013). The observed decrease in the number of archaea might be explained by an increase in the oxygenation and/or redox potential in the cecum, although we were not able to detect a direct effect on the redox measures with the method applied here. It could also be the case that essential bacteria-archaea interactions are disrupted by amoxicillin treatment, explaining the reduced abundance of archaea.

In conclusion, the results obtained in this study elucidate the effects of amoxicillin on redox potential and antioxidant capacity in the gut of rats. In contrast to previous reports, our results do not indicate that antibiotic treatment (amoxicillin) leads to an increase of the luminal redox potential in the gut, despite affecting luminal pH, bacterial composition, archaeal load, and microbial metabolism.

## Supplementary Material

fiaf003_Supplemental_File
