# Correction to: Oral amoxicillin treatment disrupts the gut microbiome and metabolome without interfering with luminal redox potential in the intestine of Wistar Han rats

**DOI:** 10.1093/femsec/fiaf070

**Published:** 2025-06-30

## Abstract

While other hyperparasitic species have been reported to control coffee rust, *Acremonium persicinum* is the first to be identified in this study.

This is a correction to: Sandra Bermúdez-Sánchez, Martin Iain Bahl, Egon Bech Hansen, Tine Rask Licht, Martin Frederik Laursen, Oral amoxicillin treatment disrupts the gut microbiome and metabolome without interfering with luminal redox potential in the intestine of Wistar Han rats, *FEMS Microbiology Ecology*, Volume 101, Issue 2, February 2025, fiaf003, https://doi.org/10.1093/femsec/fiaf003.

In the originally published version of this manuscript, the images of Figure 4 and Figure 5 were inadvertently transposed.

Additionally, in the legend belonging to Figure 4, the descriptions of panels (A), (B) and (C) were incorrectly ordered.

These errors have been corrected in the article.

