# Supplementary material for: Oral amoxicillin treatment disrupts the gut microbiome and metabolome without interfering with luminal redox potential in the intestine of Wistar Han rats
Source: FEMS Microbiol Ecol. 2025 Jan 8;101(2):fiaf003. doi: 10.1093/femsec/fiaf003 (PMC11775830; doi:10.1093/femsec/fiaf003)
Supplement: fiaf003_Supplemental_File [file fiaf003_supplemental_file.docx]

**Supplementary material for “*Oral amoxicillin treatment disrupts the gut microbiome and metabolome without interfering with luminal redox potential in the intestine of Wistar Han rats*”**


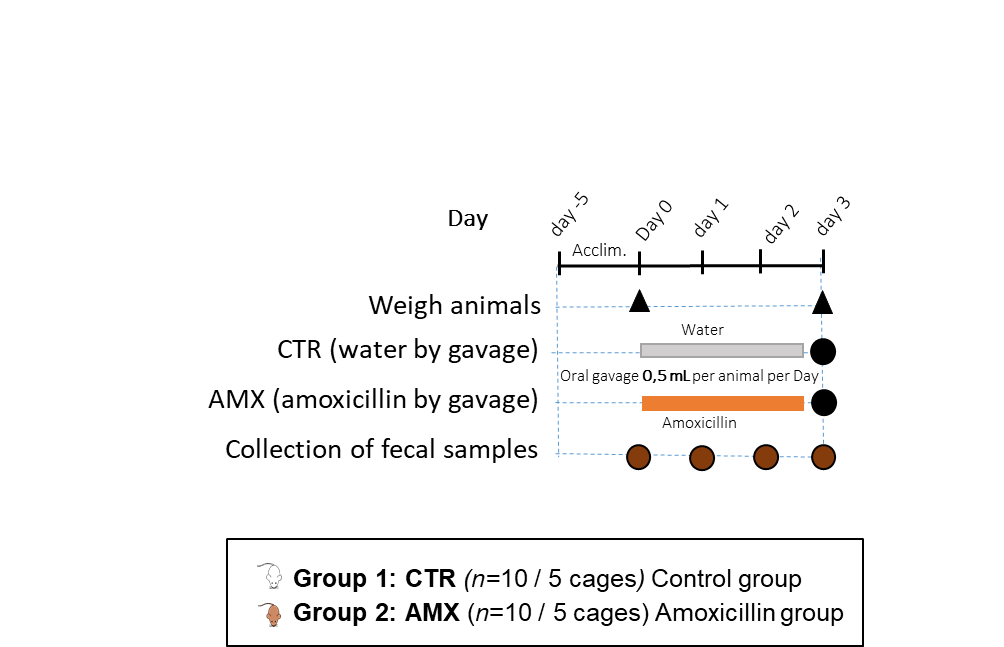


**B**

**A**

**Supplementary Figure 1. A) Follow-up animal study design to investigate acute effects on amoxicillin on redox potential in the rat’s gut.** The animal experiment started on day 0 after 5 days of acclimatization period. On day 0, amoxicillin (60mg/ml) was given to group 2 (AMX) by oral gavage, while water was given to group 1 (CTR), for three consecutive days. Black triangles indicate when the animals were weighted. Brown circles indicate the time points where a faecal sample was collected for each animal. Solid black circles show when animals from each group were dissected, caecal samples were collected for further analyses and redox potential was measured inside the cecum of all rats. **B) Effect of 3-day oral amoxicillin on antioxidant capacity, caecal redox potential, and weight.** Caecal weight showed a greater size of the cecum of the rats that received amoxicillin treatment (Mann-Whitney test, p<0.001), however**,** no significant differences were observed after T-test was used to compare the percentage of scavenging capacity or caecal redox potential. P *<0.05, P**<0.01, P***<0.001.

**Supplementary Figure 2.** DPPH assay standard curve with trolox as an antioxidant. Standard curve was performed to calculate the antioxidant capacity of the caecal content of the first animal study. Linear regression shows the percentage of DPPH scavenged by different dilutions of trolox, which was used as a reference to calculate the antioxidant capacity of the caecal samples at different time points.


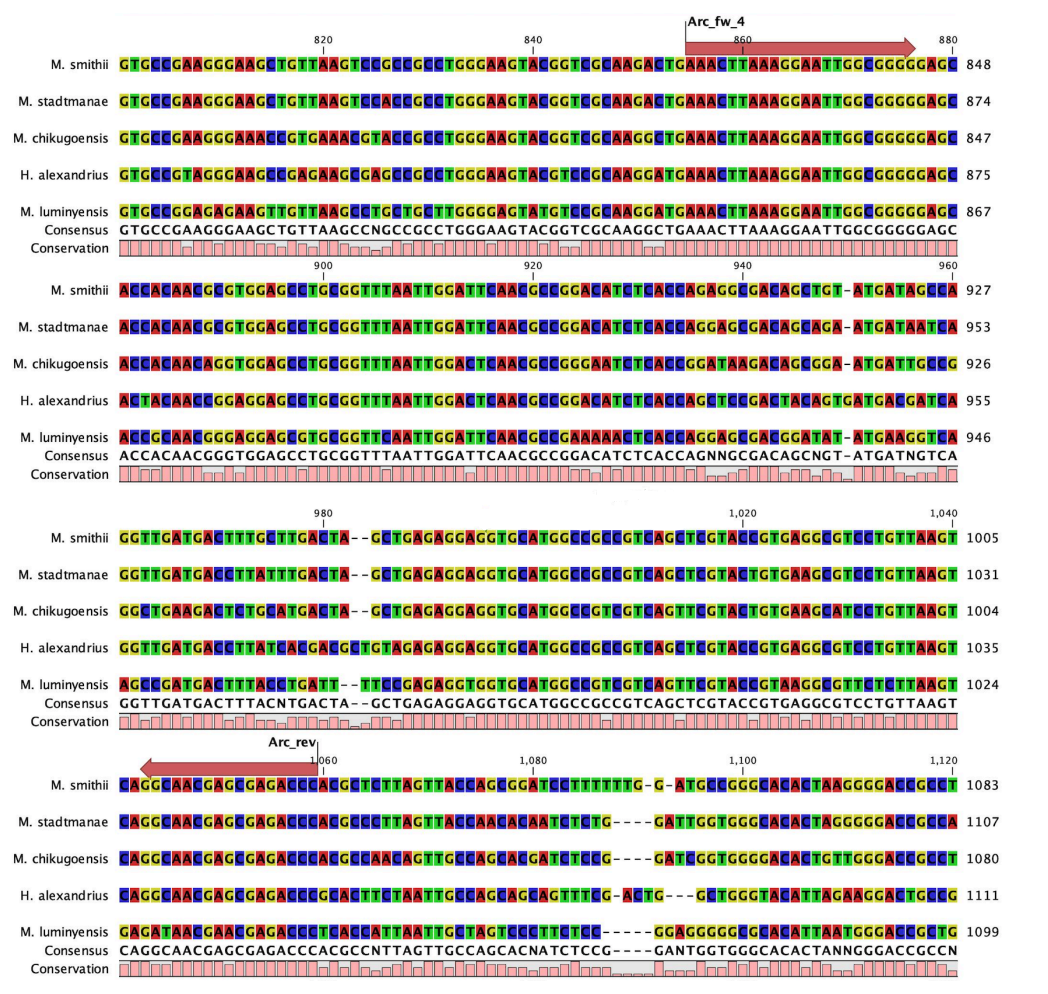


**Supplementary Figure 3.** Alignment of most abundant intestinal archaea strains and coverage of archaeal primers to test them in silico. Arc_fw_4 and Arc_rev showed good coverage for the most abundant archaea species in the intestine.

**A**

**Supplementary Figure 4.** Water consumption during the animal study. No differences were observed for water consumed by the animals at any time point by using Kruskal-Wallis test.

**Supplementary Figure 5.** Bar diagram plot of the principal orders and phyla in the three different groups of rat’s caecal content.

**Supplementary Figure 6.** SCFAs concentration (µmol/g) in rat’s caecal samples. One-way ANOVA and Tukey’s tests or the non-parametric Kruskal-Wallis and Dunn’s tests were used to compare the differences in the concentration of the SCFAs analysed by GC-MS in the caecal samples of the animals at the three different time points. The LOD/2 value was used to plot the samples that were under the detection limit.

**Supplementary Figure 7.** Food amino acids concentration (µmol/g) in rat’s caecal samples. One-way ANOVA and Tukey’s tests or the non-parametric Kruskal-Wallis and Dunn’s tests were used to compare the differences in the concentration of the amino acids analysed by GC-MS in the caecal samples of the animals at the three different time-points. The LOD/2 value was used to plot the samples that were under the detection limit.

**
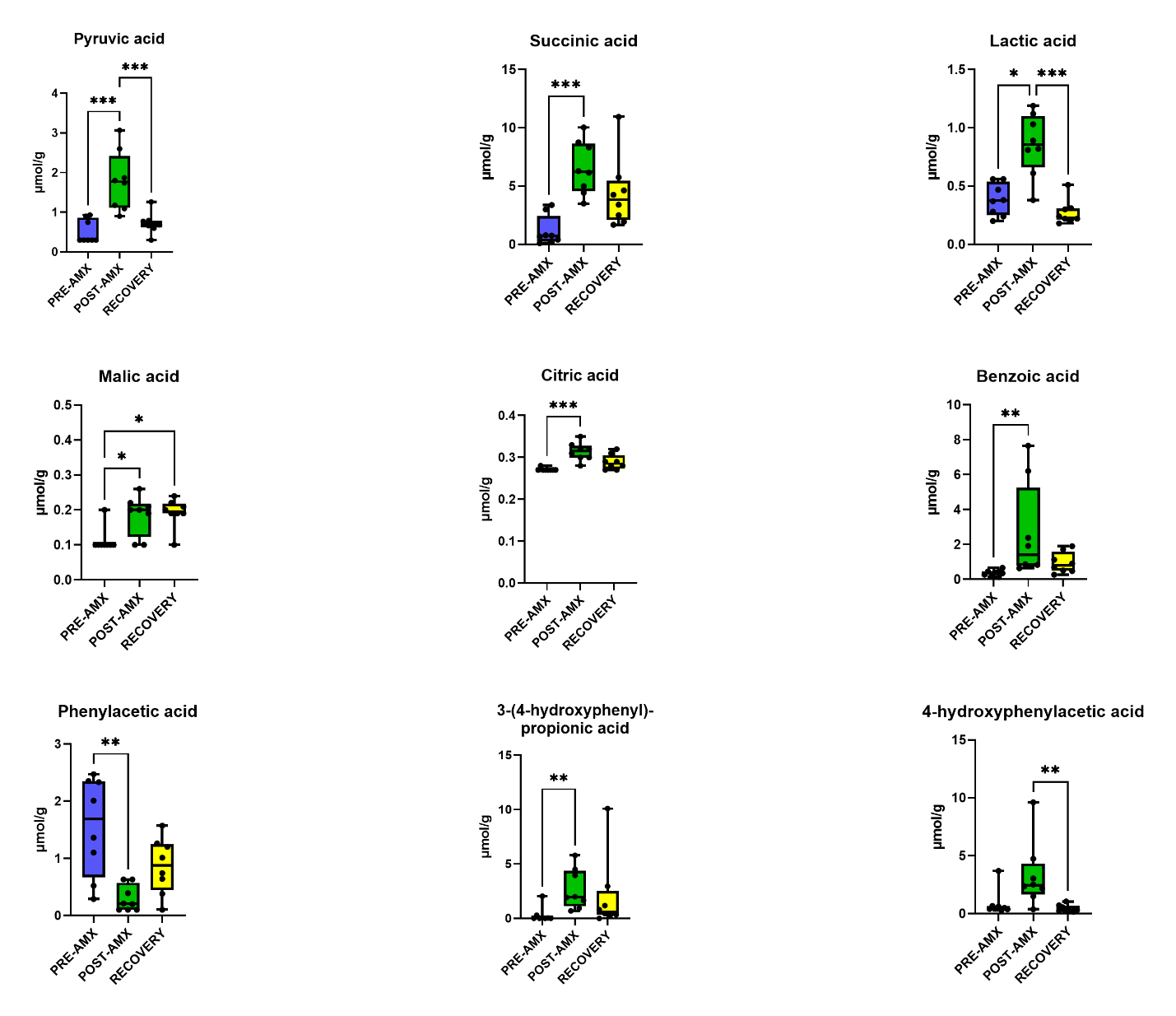
**

**Supplementary Figure 8.** Organic amino acids concentration (µmol/g) in rat’s caecal samples. One-way ANOVA and Tukey’s tests or the non-parametric Kruskal-Wallis and Dunn’s tests were used to compare the differences in the concentration of the organic acids analysed by GC-MS in the caecal samples of the animals at the three different time points. The LOD/2 value was used to plot the samples that were under the detection limit.

**Supplementary Figure 9.** Relative abundances of A) *Lachnospiraceae* and B) *Ruminococcaceae* in rat’s caecal samples. Kruskal-Wallis and Dunn’s tests were used to compare the differences in the relative abundances between groups.
